# Supplementary material for: Periodontitis and pre-eclampsia among pregnant women in Rwanda: A case-control study
Source: PLoS One. 2024 Oct 14;19(10):e0312103. doi: 10.1371/journal.pone.0312103 (PMC11472930; doi:10.1371/journal.pone.0312103)
Supplement: S1 Checklist — (DOC) [file pone.0312103.s001.doc]

STROBE Statement—Checklist of items that should be included in reports of ***case-control studies***

|  | Item No | Recommendation |
| --- | --- | --- |
| **Title and abstract** | 1 | (*a*) Indicate the study’s design with a commonly used term in the title or the abstract  **Yes check Title on page No1** |
|  |  | (*b*) Provide in the abstract an informative and balanced summary of what was done and what was found  **Yes check page No 1&2** |
| Introduction | | |
| Background/rationale | 2 | Explain the scientific background and rationale for the investigation being reported. **Yes, see on page No 2, 3&4** |
| Objectives | 3 | State specific objectives, including any prespecified hypotheses.  **No, See on page No 4** |
| Methods | | |
| Study design | 4 | Present key elements of study design early in the paper  **Yes refer on the page No 4** |
| Setting | 5 | Describe the setting, locations, and relevant dates, including periods of recruitment, exposure, follow-up, and data collection  **Yes refer on the page No 5** |
| Participants | 6 | (*a*) Give the eligibility criteria, and the sources and methods of case ascertainment and control selection. Give the rationale for the choice of cases and controls  **Refer on the page No 5** |
|  |  | (*b*) For matched studies, give matching criteria and the number of controls per case **No, it was unmatched study. See page No 4** |
| Variables | 7 | Clearly define all outcomes, exposures, predictors, potential confounders, and effect modifiers. Give diagnostic criteria, if applicable  **Refer on the page No 4,5&6** |
| Data sources/ measurement | 8* | For each variable of interest, give sources of data and details of methods of assessment (measurement). Describe comparability of assessment methods if there is more than one group Population, how measured,  **Refer to page No 4, 5&6** |
| Bias | 9 | Describe any efforts to address potential sources of bias  **Yes refer 4&5** |
| Study size | 10 | Explain how the study size was arrived at **156**  **Yes, Refer page No 4** |
| Quantitative variables | 11 | Explain how quantitative variables were handled in the analyses. If applicable, describe which groupings were chosen and why  **Refer page No 6**  **Grouping N/A** |
| Statistical methods | 12 | (*a*) Describe all statistical methods, including those used to control for confounding **Yes,** **Refer to page No 6** |
|  |  | (*b*) Describe any methods used to examine subgroups and interactions  **Yes, Refer to Page No 6** |
|  |  | (*c*) Explain how missing data were addressed **N/A** |
|  |  | (*d*) If applicable, explain how matching of cases and controls was addressed **N/A** |
|  |  | (*e*) Describe any sensitivity analyses **N/A** |
| Results | | |
| Participants | 13* | (a) Report numbers of individuals at each stage of study—eg numbers potentially eligible, examined for eligibility, confirmed eligible, included in the study, completing follow-up, and analysed  **Yes, refer Figure 1 page No20** |
|  |  | (b) Give reasons for non-participation at each stage  **Yes, refer page No 5** |
|  |  | (c) Consider use of a flow diagram  **Yes, refer page No 20** |
| Descriptive data | 14* | (a) Give characteristics of study participants (eg demographic, clinical, social) and information on exposures and potential confounders  **Refer page No 7,18&19** |
|  |  | (b) Indicate number of participants with missing data for each variable of interest **N/A** |
| Outcome data | 15* | Report numbers in each exposure category, or summary measures of exposure  **Refer tables page No 18&19** |
| Main results | 16 | (*a*) Give unadjusted estimates and, if applicable, confounder-adjusted estimates and their precision (eg, 95% confidence interval). Make clear which confounders were adjusted for and why they were included  **See results Page No 7, 8 and tables pages 18 &19** |
|  |  | (*b*) Report category boundaries when continuous variables were categorized  **N/A** |
|  |  | (*c*) If relevant, consider translating estimates of relative risk into absolute risk for a meaningful time period  **N/A** |

| Other analyses | 17 | Report other analyses done—eg analyses of subgroups and interactions, and sensitivity analyses **N/A** |
| --- | --- | --- |
| Discussion | | |
| Key results | 18 | Summarise key results with reference to study objectives  **Refer to page No 8, 9&10** |
| Limitations | 19 | Discuss limitations of the study, taking into account sources of potential bias or imprecision. Discuss both direction and magnitude of any potential bias  **Refer page No 10** |
| Interpretation | 20 | Give a cautious overall interpretation of results considering objectives, limitations, multiplicity of analyses, results from similar studies, and other relevant evidence  **Refer to page No 8, 9&10** |
| Generalizability | 21 | Discuss the generalizability (external validity) of the study results  **Yes, Refer page No 10** |
| Other information | | |
| Funding | 22 | Give the source of funding and the role of the funders for the present study and, if applicable, for the original study on which the present article is based  **Refer to page No 11** |

*Give information separately for cases and controls.

**Note:** An Explanation and Elaboration article discusses each checklist item and gives methodological background and published examples of transparent reporting. The STROBE checklist is best used in conjunction with this article (freely available on the Web sites of PLoS Medicine at http://www.plosmedicine.org/, Annals of Internal Medicine at http://www.annals.org/, and Epidemiology at http://www.epidem.com/). Information on the STROBE Initiative is available at http://www.strobe-statement.org.
