# Supplementary material for: Periodontitis and pre-eclampsia among pregnant women in Rwanda: A case-control study
Source: PLoS One. 2024 Oct 14;19(10):e0312103. doi: 10.1371/journal.pone.0312103 (PMC11472930; doi:10.1371/journal.pone.0312103)
Supplement: Attachment — Submitted filename: Point-by-point response to editor and reviewers 12.09.24.docx [file pone.0312103.s002.docx]

**AUTHOR RESPONSE TO EDITOR AND REVIEWERS**

We wish to express our sincere appreciation to the editor and reviewers for their insightful comments. We have done our best to address all the issues raised and, we hope that the final version of this manuscript is much improved due to their comments.

| **Editor’s Comments** | **Page and line** |  |
| --- | --- | --- |
| 1. The introduction provides a broad overview of the literature but lacks a critical evaluation of the existing studies. Some of the references used are outdated or lack relevance to the current study's geographical context in the introduction and discussion sections. | **Page 3-5**  **Line 6, 97, 113, 117.** | **Response to Editor’s Comment**  Thank you for your valuable feedback. We have revised the introduction to address the critical evaluation of existing studies and ensure relevance to the current geographical context as follows:  ***Introduction***  *Pre-eclampsia is a major complication of pregnancy characterized by gestational hypertension and associated with significant maternal and fetal risks, including impaired fetal development, maternal morbidity and mortality[1]. Globally, the prevalence of pre-eclampsia ranges from 2% to 8% of pregnancies [2,3], with a prevalence of 4.1% in developing countries like Sub-Saharan Africa [4]. In Rwanda, recent studies have reported a prevalence of 2.3% in Kigali teaching hospitals [5] and approximately 3% in rural areas [6], highlighting the ongoing challenge posed by this condition.*  *According to the American College of Obstetrics and Gynecology (ACOG), pre-eclampsia is defined as hypertension (≥ 140/90 mm Hg) and proteinuria (300 mg or more per 24-hour urine collection) or, in the absence of proteinuria, new-onset hypertension with associated complications, such as thrombocytopenia, renal insufficiency, impaired liver function, pulmonary edema, severe headache unresponsive to medication, or visual disturbances [3,7]. This condition typically manifests after 20 weeks of gestation [3,7], underscoring the importance of early detection and management.*  *Pre-eclampsia contributes to approximately 50,000 maternal deaths globally each year [8]. In low- and middle-income countries, it is responsible for 10-15% of maternal deaths [9], with Rwanda experiencing a rate of 13% [10]. The condition's multifactorial etiology includes factors such as endothelial dysfunction, systemic inflammation, and various risk factors like primiparity, family history, uterine malformation, pre-existing hypertension, diabetes, obesity, placental abnormalities, renal disease [11,12]. Recent research has highlighted the role of systemic inflammation and endothelial dysfunction in the pathogenesis of pre-eclampsia [13–15]. Systemic inflammation, often exacerbated conditions such as periodontitis, has been proposed as a key mechanism contributing to the development of pre-eclampsia [14,16].*  *Periodontitis****,*** *a chronic inflammatory disease caused by localized bacterial infections, results in the destruction of periodontal tissues and can lead to tooth loss [17,18]. Globally, severe periodontitis affects approximately 616 million people [19,20], with a higher prevalence of 13.5 % reported in central Sub-Saharan Africa [21]. In Rwanda, a national oral health survey found a high prevalence of periodontitis in general population, with 60% of people experiencing dental calculus and 34.2% having dental debris [22]. Furthermore, periodontitis is found in up to 40% of pregnant women [23], and a growing body of evidence suggests a compelling link between periodontitis and adverse pregnancy outcomes, including pre-eclampsia [24–27]. Studies have reported associations with adverse pregnancy outcomes such as preterm births, low birth weight and pre-eclampsia [28–30]. For instance, research in Korea found a significant association between periodontitis and an increased risk of pre-eclampsia [31], while studies in Tanzania revealed a substantial association with high odds of pre-eclampsia (aOR=4.12; 95% CI: 2.20-7.90) [32].*  *The mechanistic relationship between periodontitis and pre-eclampsia involves several key pathways. Chronic systemic inflammation resulting from periodontitis may exacerbate the inflammatory processes involved in pre-eclampsia. Periodontal disease can lead to elevated levels of inflammatory markers such as C-reactive protein (CRP), interleukin-6 (IL-6), and tumor necrosis factor-alpha (TNF-α), which may contribute to endothelial dysfunction and impaired placental blood flow, thereby increasing the risk of pre-eclampsia [33,34]. This connection underscores the need for further research to explore these inflammatory markers and their role in the pathogenesis of pre-eclampsia.* *Systematic review from 30 studies confirms that pre-eclampsia is a significant risk factor for periodontitis, and even suggests that is more pronounced in lower-middle-income countries [35,36]*  *Despite the growing body of evidence, most research on the relationship between periodontal disease and pre-eclampsia has been in developed countries, creating a significant knowledge gap for low and middle income countries. In Rwanda, where both pre-eclampsia and periodontal disease are prevalent, there is a need for localized studies to better understand this association. This study aims to investigate the association between periodontitis and pre-eclampsia in Rwanda. By doing so, it seeks to provide valuable insights into how these conditions interact in the context of Rwanda, contributing to improved health outcomes and advancing of knowledge in this critical area.* |
| 2. The explanation of the sample size calculation using G*Power is incomplete.  The effect size of 0.50 is mentioned, but it is unclear what this value represents. Typically, in case-control studies, the effect size might refer to the odds ratio (OR) expected between exposure (e.g., periodontitis) and the outcome (e.g., pre-eclampsia). It would be helpful to include more details on the effect size and the assumptions made for the calculation. | **Page 5, 6**  **Line 140-149** | **Response to Reviewer’s Comments**  Thank you for your valuable feedback. We have revised the Methods section to address your concerns regarding the sample size calculation and the effect size as follows:  *To determine the sample size, we utilized G* Power Software, aiming for a significance level (α\alpha) of 0.05 and a target power of 85%, which is a common standard for many studies. to determine the necessary number of participants. The effect size was set at 0.50, representing a medium to large odds ratio (OR) of approximately 2.61. Based on this effect size and power level, the required total sample size was 156 participants, with 52 women with pre-eclampsia (case) and 104 women without pre-eclampsia (control) [35], (****Figure 1****).* |
| 3. There is a limited discussion with regards to other studies findings. Add other studies results in discussion section. | Page 15-19  Line 313-314,  327, 328, 351, 363, 425 | Thank you for you very insightful comment, we have significantly improved the discussion section as follows:  ***Discussion***  *The findings of this study support the hypothesis that periodontitis is associated with pre-eclampsia in two selected referral hospitals in Rwanda. Our observation of a notably higher prevalence of periodontitis (90.4%), including moderate/ severe periodontitis cases, among women with pre-eclampsia aligns with several global studies. For instance, a study done in India found a similar association, where women with periodontitis had a significantly higher risk of developing pre-eclampsia standing at 90% [41]. This pattern is also observed in studies from Iran at 98% [15] and Turkey at 74.3% [42], reinforcing the potential global relevance of this association.*  *The prevalence of periodontitis in our study population significantly exceeded that of the general adult population in Rwanda (39.6 %), [43].This elevated prevalence among pre-eclamptic women mirrors findings from studies in the USA, where pregnant women with periodontitis were found to have higher rates of pre-eclampsia compared to those without periodontitis[16]. Similarly, a Tanzanian study demonstrated a strong association between periodontal disease and adverse pregnancy outcomes, including pre-eclampsia [32].*  *In contrast, some studies have reported conflicting results. For example, a study conducted in Norway found no significant association between periodontitis and pre-eclampsia after adjusting for confounders [44]. Additionally, a study from Japan reported similar findings, where the link between periodontal disease and pre-eclampsia was not statistically significant [45]. These discrepancies could be due to differences in study populations, periodontal disease definitions, or variations in healthcare access and practices across different countries.*  *Our multivariate logistical regression analysis was adjusted for potential confounders such as age, alcohol consumption, primiparity, edema, and headache. The resulting odds ratio of 3.85, indicates that women with moderate/severe periodontitis are over three times more likely to develop pre-eclampsia, which is consistent with a meta-analysis that reported an overall odds ratio of 3.18 [46]. Interestingly, this meta-analysis found that the association was even stronger in studies conducted in low- and middle-income countries (OR= 6.70), [46], underscoring the importance of context-specific research.*  *In terms of clinical indicators, our study found significantly higher mean periodontal pocket depth (PPD) and clinical attachment loss (CAL) in the pre-eclamptic group, which aligns with the findings from studies in Brazil and India. In Brazil, researchers observed that pre-eclamptic women had greater PPD and CAL compared to their non-pre-eclamptic counterparts [47]. Similarly, an Indian study highlighted significant differences in periodontal measurements between pre-eclamptic and non-pre-eclamptic women, further supporting the association between periodontal health and pregnancy outcomes [48]. These findings suggest that the severity of periodontal disease may be a critical factor in the development of pre-eclampsia.*  *Univariate analysis in our study identified association between pre-eclampsia and various risk factors, including age, study location, alcohol consumption during pregnancy, visit to dental services, edema, headache, primiparity, pregnant women’s weight and visual disturbances.*  *The final multivariate logistic regression confirmed age, study location, edema and headache as significant risks factors for pre-eclampsia. These results are consistent with findings from studies done in Chile and Bangladesh, which identified similar risk factors for pre-eclampsia, including maternal age and pre-existing hypertension [49,50]. Additionally, studies in Sweden and South Africa emphasized the role of socio-economic factors in pre-eclampsia risk, [51,52] suggesting that future research in Rwanda should also consider these variables.*  *Interestingly, our study found a significant association between dental service visits and pre-eclampsia, which contrasts with findings from Boggers et al, that reported no significant link between dental visits and pre-eclampsia [53]. This difference might be explained by varying access to dental care and differences in dental service utilization during pregnancy across different healthcare systems. Furthermore, our study did not find an association between tobacco consumption and pre-eclampsia, a finding that diverges from studies in Sweden and the USA, which reported smoking as a significant risk factor for pre-eclampsia [47,54].*  *Potential mechanisms for the link between periodontitis and pre-eclampsia include the translocation of periodontal pathogens to the placenta, leading to localized inflammation and oxidative stress, which may contribute to endothelial dysfunction—a key feature of pre-eclampsia [46]. This theory is supported by studies that detected periodontal bacteria in placental tissues of pre-eclamptic women, suggesting a direct pathogenic role [46,55] . Moreover, the hypothesis that repeated bacteremia exposes decidual tissues to periodontal bacteria, has been proposed as another pathway, although the exact mechanism remain to be fully elucidated [15,49]. Despite these plausible explanations, the multifactorial nature of both conditions necessitates further research to clarify these pathways and determine whether improving periodontal health can reduce pre-eclampsia risk.*  ***Strength and limitations of our study***  *Our study is the first to explore association between periodontitis and pre-eclampsia among pregnant women in Rwanda, offering valuable insights into this important public health issue. However, the small sample size and omission of certain socio-demographic characteristics may limit the generalizability of our findings. Future research should aim to collect individual-level data on socioeconomic factors to deepen our understanding. Additionally, the absence of dental radiographs due to the safety concern, a limitation noted in similar studies [38–40], restricted our ability to assess of bone loss radiographically and provide a universally accepted diagnosis of periodontal disease. While we adjusted for known confounders in our multivariate logistic regression model, residual confounding may still exist due to the lack of detailed endothelial function measures or accurate quantifications of placental insufficiency. These limitations highlight the need for future studies to include larger sample sizes, comprehensive socio-economic data, additional inflammatory markers, and advanced diagnostic methods to strengthen the observed associations and provide a more robust understanding of the relationship between periodontitis and pre-eclampsia.*  ***Conclusion***  *This study establishes a robust association between periodontitis and pre-eclampsia among pregnant women in Rwanda, even after accounting for potential confounders. Our findings indicate that pregnant women with periodontitis are approximately three times more likely to develop pre-eclampsia compared to their counterparts. This significant association underscores the impact of periodontal health on maternal outcomes and highlights the importance of integrating oral health care into prenatal care practices, as interventions to manage periodontitis once pregnancy may be too late; non-surgical therapy for example does not reduce adverse pregnancy outcomes [57].*  *Despite some limitations, our research contributes valuable insights into the association between periodontal disease and pre-eclampsia. The evidence suggests that improving oral health could be a viable strategy to reduce the risk of pre-eclampsia, emphasizing the need for preventive interventions. The findings have important implications for maternal and fetal health, particularly in resource-limited settings where access to comprehensive dental care may be limited. Future research should address regional variations in healthcare access, socio-economic factors, and the definitions of periodontal disease to better understand these relationships.* |
| **General comments** |  |  |
| 1. If the authors have adequately addressed your comments raised in a previous round of review and you feel that this manuscript is now acceptable for publication, you may indicate that here to bypass the “Comments to the Author” section, enter your conflict of interest statement in the “Confidential to Editor” section, and submit your "Accept" recommendation.  Reviewer #2: All comments have been addressed  Reviewer #3: (No Response)  Reviewer #4: All comments have been addressed |  | We would like to express our gratitude to the reviewers for their valuable feedback and careful consideration of our manuscript. We are pleased to note that Reviewers have acknowledged that all comments have been adequately addressed. |
| 2. Is the manuscript technically sound, and do the data support the conclusions?  The manuscript must describe a technically sound piece of scientific research with data that supports the conclusions. Experiments must have been conducted rigorously, with appropriate controls, replication, and sample sizes. The conclusions must be drawn appropriately based on the data presented.  Reviewer #2: Yes  Reviewer #3: Partly  Reviewer #4: (No Response) |  | We acknowledge the feedback provided by the reviewers. Reviewer #2 has confirmed that the manuscript is technically sound, while Reviewer #3 has indicated partial agreement. We would appreciate the concerns from Reviewer #3 and we have addressed them accordingly. ***(See below responses to review#3)*** |
| 3. Has the statistical analysis been performed appropriately and rigorously?  Reviewer #2: Yes  Reviewer #3: No  Reviewer #4: (No Response) |  | We acknowledge Reviewer #2's approval feedback and we have addressed the Reviewer #3's concerns. ***(See below responses to review#3)*** |
| 4. Have the authors made all data underlying the findings in their manuscript fully available?  Reviewer #2: Yes  Reviewer #3: Yes  Reviewer #4: (No Response) |  | We are pleased that both Reviewer #2 and Reviewer #3 confirm that all underlying data have been made fully available. |
| 5. Is the manuscript presented in an intelligible fashion and written in standard English?  Reviewer #2: Yes  Reviewer #3: Yes  Reviewer #4: (No Response) |  | We are glad that both Reviewer #2 and Reviewer #3 have affirmed that the manuscript is presented intelligibly and written in standard English. |
| **Reviewer #2: Dear, Authors**  It’s my great pleasure to be assigned as a reviewer of this research that focuses on “Periodontitis and Pre-eclampsia among Pregnant Women in Rwanda: A Case-Control Study,” which has great public health importance.  I found that you incorporated all the suggestions and comments I have given before. I am satisfied with all your suggestions. Still, there might be minor errors in a whole document. So you have to work on that to make your paper intelligible to the scientific community. |  | Thank you for your positive feedback. We have reviewed the manuscript for any remaining minor errors to ensure clarity for the scientific community. |
| **Reviewer #3: Periodontitis and Pre-eclampsia among Pregnant Women in Rwanda: A Case-Control Study.** |  |  |
| While the manuscript is very written as it is, here are a few concerns that needs to be addressed.  What is the specific prevalence of periodontitis in Rwanda, while the author provides reference for sub-Saharan African there is none specific to Rwanda.  Moreover, Authors did not provide a convincing fine mechanistic relationship between Preeclampsia and Periodontitis in the manuscript. Thus, it is easy to presume that if oral hygiene is a public health concern in Rwanda, then finding a high prevalence of periodontitis in women with preeclampsia is also plausible | **Page 3-5**  **Line 6, 97, 113, 117.** | **Response to Reviewer’s Comment**  Thank you for highlighting the need for specific prevalence data on periodontitis in Rwanda. We have revised the introduction to include precise information on the prevalence of periodontitis specific to Rwanda. Additionally, we have revised the introduction to address the mechanistic relationship between pre-eclampsia and periodontitis and the suggestion to analyze additional inflammatory markers. The introduction was thoroughly revised as follows:  ***Introduction***  *Pre-eclampsia is a major complication of pregnancy characterized by gestational hypertension and associated with significant maternal and fetal risks, including impaired fetal development, maternal morbidity and mortality[1]. Globally, the prevalence of pre-eclampsia ranges from 2% to 8% of pregnancies [2,3], with a prevalence of 4.1% in developing countries like Sub-Saharan Africa [4].* ***In Rwanda, recent studies have reported a prevalence of 2.3% in Kigali teaching hospitals [5] and approximately 3% in rural areas [6], highlighting the ongoing challenge posed by this condition.***  *According to the American College of Obstetrics and Gynecology (ACOG), pre-eclampsia is defined as hypertension (≥ 140/90 mm Hg) and proteinuria (300 mg or more per 24-hour urine collection) or, in the absence of proteinuria, new-onset hypertension with associated complications, such as thrombocytopenia, renal insufficiency, impaired liver function, pulmonary edema, severe headache unresponsive to medication, or visual disturbances [3,7]. This condition typically manifests after 20 weeks of gestation [3,7], underscoring the importance of early detection and management.*  *Pre-eclampsia contributes to approximately 50,000 maternal deaths globally each year [8]. In low- and middle-income countries, it is responsible for 10-15% of maternal deaths [9], with Rwanda experiencing a rate of 13% [10]. The condition's multifactorial etiology includes factors such as endothelial dysfunction, systemic inflammation, and various risk factors like primiparity, family history, uterine malformation, pre-existing hypertension, diabetes, obesity, placental abnormalities, renal disease [11,12]. Recent research has highlighted the role of systemic inflammation and endothelial dysfunction in the pathogenesis of pre-eclampsia [13–15]. Systemic inflammation, often exacerbated conditions such as periodontitis, has been proposed as a key mechanism contributing to the development of pre-eclampsia [14,16].*  *Periodontitis****,*** *a chronic inflammatory disease caused by localized bacterial infections, results in the destruction of periodontal tissues and can lead to tooth loss [17,18]. Globally, severe periodontitis affects approximately 616 million people [19,20], with a higher prevalence of 13.5 % reported in central Sub-Saharan Africa [21].* ***In Rwanda, a national oral health survey found a high prevalence of periodontitis in general population, with 60% of people experiencing dental calculus and 34.2% having dental debris [22].*** *Furthermore, periodontitis is found in up to 40% of pregnant women [23], and a growing body of evidence suggests a compelling link between periodontitis and adverse pregnancy outcomes, including pre-eclampsia [24–27]. Studies have reported associations with adverse pregnancy outcomes such as preterm births, low birth weight and pre-eclampsia [28–30]. For instance, research in Korea found a significant association between periodontitis and an increased risk of pre-eclampsia [31], while studies in Tanzania revealed a substantial association with high odds of pre-eclampsia (aOR=4.12; 95% CI: 2.20-7.90) [32].*  ***The mechanistic relationship between periodontitis and pre-eclampsia involves several key pathways. Chronic systemic inflammation resulting from periodontitis may exacerbate the inflammatory processes involved in pre-eclampsia. Periodontal disease can lead to elevated levels of inflammatory markers such as C-reactive protein (CRP), interleukin-6 (IL-6), and tumor necrosis factor-alpha (TNF-α), which may contribute to endothelial dysfunction and impaired placental blood flow, thereby increasing the risk of pre-eclampsia [33,34]. This connection underscores the need for further research to explore these inflammatory markers and their role in the pathogenesis of pre-eclampsia. Systematic review from 30 studies confirms that pre-eclampsia is a significant risk factor for periodontitis, and even suggests that is more pronounced in lower-middle-income countries [35,36]***  *Despite the growing body of evidence, most research on the relationship between periodontal disease and pre-eclampsia has been in developed countries, creating a significant knowledge gap for low and middle income countries. In Rwanda, where both pre-eclampsia and periodontal disease are prevalent, there is a need for localized studies to better understand this association. This study aims to investigate the association between periodontitis and pre-eclampsia in Rwanda. By doing so, it seeks to provide valuable insights into how these conditions interact in the context of Rwanda, contributing to improved health outcomes and advancing of knowledge in this critical area.* |
| Perhaps as the author relate this two conditions to inflammation- additional inflammatory markers could have been analyzed to provide novelty, a more solid association, and not only statistical inference.  The stated study limitations are within the remit of the study and authors should have performed these. | **Page 18**  **Line 397-414** | Thank you for your thoughtful feedback. In response to your comments, we have revised the "Strength and Limitations of Our Study" section to address your concerns. We acknowledged the need for additional inflammatory markers to enhance the novelty and solidify the association between periodontitis and pre-eclampsia. We also recognized the study's limitations, such as the small sample size and the lack of dental radiographs, and suggested that future research should address these gaps by including larger sample sizes, comprehensive data, and advanced diagnostic methods. We believe these revisions strengthen our manuscript and provide a clearer direction for future studies. The section was improved as follows:  *Our study is the first to explore association between periodontitis and pre-eclampsia among pregnant women in Rwanda, offering valuable insights into this important public health issue. However, the small sample size and omission of certain socio-demographic characteristics may limit the generalizability of our findings. Future research should aim to collect individual-level data on socioeconomic factors to deepen our understanding. Additionally, the absence of dental radiographs due to the safety concern, a limitation noted in similar studies [38–40], restricted our ability to assess of bone loss radiographically and provide a universally accepted diagnosis of periodontal disease. While we adjusted for known confounders in our multivariate logistic regression model, residual confounding may still exist due to the lack of detailed endothelial function measures or accurate quantifications of placental insufficiency. These limitations highlight the need for future studies to include larger sample sizes, comprehensive socio-economic data, additional inflammatory markers, and advanced diagnostic methods to strengthen the observed associations and provide a more robust understanding of the relationship between periodontitis and pre-eclampsia.* |
| Basic information on several factors used the model analysis have not been previously present in the manuscript. This should be presented and compared in both cohort before the logistic regression analysis. Information on tobacco consumption in both cohort is not evident. Information on oral hygiene is also not provided in the manuscript. These are important to contextualize the findings.  Although authors have indicated that Both conditions share many risk factors such as smoking, alcohol consumption, diabetes, and maternal age among others, some of these risk factors were not assessed in the women. or were not documented in this manuscript. | Page 9  Line 251-263 | Thank you for your insightful comments. We have addressed your concerns by including basic information on several factors that were previously missing from the manuscript. Specifically, we have added data on tobacco consumption and oral hygiene for both the non-pre-eclamptic and pre-eclamptic cohorts. These details are now presented and compared before the logistic regression analysis to better contextualize the findings. The information was added in the manuscript results (both text and table) for better clarification: ***Socio-demographic Characteristics*** *This study included 156 pregnant women, comprising 52 women with pre-eclampsia and 104 women without the condition. The mean age of all participants was 30.4 years (SD = 4.9), with significant age differences observed between non-pre-eclamptic (29.6 years) and pre-eclamptic (32.0 years) groups (P = 0.01). The gestational age averaged 30.7±19.6 weeks across the cohort.* *Regarding pregnancy and health history, 17.9% of participants were in their first pregnancy, 75% had 2-4 pregnancies and 7.1% had more than four pregnancies (Table 1). The average weight of women in pre-eclamptic group was significantly higher than in the non-pre-eclamptic group (74.5±9.2 kg versus 68.5±6.3 kg).* ***A majority (79.5%) attended fewer than four antenatal care visits. Additionally, 8.7% of the non-pre-eclamptic group and 25.0% of the pre-eclamptic group reported alcohol consumption (P = 0.015).*** ***Regarding smoking status, 2.8% of non-pre-eclamptic women were smokers compared to none in the pre-eclamptic group (P = 0.358). Although smoking rates were low, this difference may warrant further investigation. Information on oral hygiene revealed that 3.8% of non-pre-eclamptic women and 13.5% of pre-eclamptic women had visited a dental service in the previous 6 months (P = 0.027). Only a small fraction of women in both groups visited for scaling teeth (2.9% vs. 9.6%, P = 0.072).*** **Table 1 Demographic characteristics, pregnancy related, dental and medical data of participants.** ***Variables*** \| ***Non-pre-eclamptic*** \| ***Pre-eclamptic*** \| ***P value***; *Age* ***[mean (SD)]*** \| *29.6(5.0)* \| *32.0(4.5)* \| *0.011*; *Gestation in weeks* ***[mean (SD)]*** \| *30.6(4.6)* \| *31.2(3.8)* \| *0.464*; *Weight for pregnant women in Kg* ***[mean (SD)]*** \| *68.5(6.3)* \| *74.5(9.2)* \| *<0.001*; *Antenatal care visits* ***[mean (SD)]*** \| *2.8(0.9)* \| *3.0(0.7)* \| *0.175*; ***Age*** \| \| \| *0.004*; *18-28* \| *47(45.2)* \| *10(19.2)* \|; *29-39* \| *54(51.9)* \| *38(73.1)* \|; *40-59* \| *3(2.9)* \| *4(7.7)* \|; ***Weight*** \| \| \| *0.855*; *50-59* \| *8(7.7)* \| *2(3.8)* \|; *60+* \| *96(92.3)* \| *50(96.2)* \|; ***Location [n (%)]*** \| \| \| *0.001*; *CHUK* \| *39(35.5)* \| *34(65.4)* \|; *RUHENGERI Hospital* \| *65(62.5)* \| *18(34.6)* \|; ***Alcohol consumption [n (%)]*** \| \| \| *0.015*; *Yes* \| *9(8.7)* \| *13(25.0)* \|; *Stopped* \| *2(1.9)* \| *2(3.8)* \|; *Non* \| *93(89.4)* \| *37(71.2)* \|; ***Smoking status [n (%)]*** \| \| \| *0.358*; *smoker* \| *3(2.8)* \| *0(0.0)* \|; *stopped* \| *100(96.2)* \| *52(100.0)* \|; *Non* \| *1(1.0)* \| *0(0.0)* \|; ***Diabetes history [n (%)]*** \| \| \| *0.013*; *Yes* \| *0(0.0)* \| *3(5.8)* \|; *No* \| *104(100.0)* \| *49(94.2)* \|; ***Other cardiovascular diseases [n (%)]*** \| \| \| *0.013*; *Yes* \| *0(0.0)* \| *3(5.8)* \|; *No* \| *104(100.0)* \| *49(94.2)* \|; ***Parity [n (%)]*** \| \| \|; *Less than one* \| *24(23.1)* \| *4(7.7)* \| *0.022*; *Between 2 and 4* \| *71(68.3)* \| *46(88.5)* \|; *Above 4* \| *9(8.7)* \| *2(3.8)* \|; ***ANC [n (%)]*** \| \| \| *0.575*; *less than 4* \| *84(80.8)* \| *40(76.9)* \|; *4 and above* \| *20(19.2)* \| *12(23.1).* \|; ***Past history of placenta abnormalities [n (%)]*** \| \| \| *0.377*; *Yes* \| *3(2.9)* \| *3(5.8)* \|; *No* \| *101(97.1)* \| *49(94.2)* \|; ***Edema [n (%)]*** \| \| \| *<0.001*; *No* \| *90(86.5)* \| *23(44.2)* \|; *Yes* \| *14(13.5)* \| *29(55.8)* \|; ***Headache [n (%)]*** \| \| \| *<0.001*; *No* \| *94(90.4)* \| *22(42.3)* \|; *Yes* \| *10(9.6)* \| *30(57.7)* \|; ***Visual disturbances [n (%)]*** \| \| \| *<0.001*; *No* \| *101(97.1)* \| *30(57.7)* \|; *Yes* \| *3(2.9)* \| *22(42.3)* \|; ***Visited dental service in previous 6 months [n (%)]*** \| \| \| *0.027*; *Yes* \| *4(3.8)* \| *7(13.5)* \|; *No* \| *100(96.2)* \| *45(86.5)* \|; ***Visited the dental service for scaling teeth [n (%)]*** \| \| \| *0.072*; *Yes* \| *3(2.9)* \| *5(9.6)* \|; *No* \| *101(97.1)* \| *47(90.4)* \| ***Abbreviations:*** *SD, standard deviation; n, frequency; %, percentage; CHUK, Centre Universitaire Hospitalier de Kigali; ANC, Antenatal care visits; Kg, Kilograms.* |
| Reviewer #4: The authors have duly addressed all the comments raised in the thesis. This has tremendously improve the manuscript and put it in acceptable state. I have no further comment for the authors. I congratulate the for taking tine to address all the comments raised in the thesis |  | Thank you for your positive feedback and for acknowledging the improvements made to the manuscript. We appreciate your support. |
